# Low-Temperature Uniaxial Orientation Effect on the Structure and Piezoelectric Properties of the Vinylidene Fluoride-Tetrafluoroethylene Copolymer Films

**DOI:** 10.3390/ijms26136309

**Published:** 2025-06-30

**Authors:** Stanislav V. Kondrashov, Evgeniya L. Buryanskaya, Aleksey S. Osipkov, Vladimir S. Kirkin, Maria V. Butina, Pavel A. Mikhalev, Dmitry S. Ryzhenko, Mstislav O. Makeev

**Affiliations:** 1 Laboratory of Technologies of Polymer Ferroelectrics, Bauman Moscow State Technical University, Moscow 105005, Russia; stasru_59@mail.ru (S.V.K.); buryanskayael@bmstu.ru (E.L.B.); osipkov@bmstu.ru (A.S.O.); 1032212037@pfur.ru (V.S.K.); masha.butina.54@mail.ru (M.V.B.); pamikhalev@bmstu.ru (P.A.M.); dsr@bmstu.ru (D.S.R.); 2 Laboratory of Physics of Oxide Ferroelectrics, National University of Science and Technology MISIS, Moscow 119049, Russia

**Keywords:** piezoelectricity, ferroelectric polymer, flexible electronics, supramolecular structure

## Abstract

This paper considers the uniaxial orientation effect on the structure and piezoelectric properties of vinylidene fluoride-tetrafluoroethylene copolymer ferroelectric films. The films were exposed to uniaxial orientation stretching in a temperature range from 20 °C to 60 °C; then, they were contact polarized under normal conditions. The temperature dependence of the electric strength was determined. The longitudinal piezoelectric coefficient *d*_33_ values were measured by the quasi-static Berlincourt method. The piezoresponse force microscopy (PFM) method was used to investigate the film domain structure before and after polarization, and the local piezoelectric coefficient values were also calculated. Phase composition was studied using differential scanning calorimetry and infrared spectroscopy with the Fourier transform. It was found that uniaxial orientation stretching contributed to an increase in the piezoelectric coefficient *d*_33_ from 5 pC/N to 16–20 pC/N. The results obtained indicate the importance of the amorphous phase contribution to the formation of the piezoelectric properties in polymeric materials.

## 1. Introduction

Despite the fact that piezoelectric ceramics remain the main material in piezoelectric engineering today, ferroelectric polymers based on polyvinylidene fluoride (PVDF) are finding increasingly wider application in science and technology [1,2,3] due to their relatively high values of pyro- and piezoresponse, chemical resistance, biocompatibility, mechanical and technological flexibility, high impact strength, and transparency [4]. Due to its low density and relatively low speed of sound propagation, the acoustic impedance of PVDF is close to that of liquid media and biological tissues. As a result, films based on PVDF demonstrate high efficiency in electroacoustic transducers (EATs) for biomedical applications. These films are used to develop sensors and devices for monitoring and ultrasonic diagnostics [5,6,7,8], such as measuring blood flow or the mechanical properties of tissues and for imaging organs or a fetus. Therapeutic and surgical applications include physiotherapy, lithotripsy, transcranial neuromodulation, and thermal or mechanical ablation of tumors [9].

PVDF-based EATs show the highest efficiency in hydrostatic (volumetric) mode when the acoustic pressure acts on the sensitive element simultaneously and uniformly from all directions [10]. The sensitivity of such an EAT, denoted as *m*_0_, is determined by the film thickness *t* and the hydrostatic piezoelectric voltage coefficient *g_h_*:

*m*_0_ = *g_h_*·*t* [V/Pa], (1)

The coefficient *g_h_* can be expressed in terms of the macroscopic piezoelectric coefficients *d*_33_, *d*_31_, and *d*_32_ as follows:

*g_h_* = (*d*_33_ + *d*_31_ + *d*_32_)/*ε*_0_*ε*′, (2)

where *ε*_0_ is the vacuum permittivity, and *ε*′ is the real part of the dielectric permittivity. For PVDF films, *g_h_* is an order of magnitude higher than in ceramic materials. However, while the electroactive properties of piezoceramics are mainly determined by their chemical composition, in such polymer ferroelectrics, the material’s structure—formed during technological processes like orientation (stretching) or annealing—has a significant impact on these properties.

According to the generally accepted assumptions of today [11,12], cooling the melt in a PVDF or its copolymers extraction leads to polymer matrix crystallization in TGTG′ conformation (α-phase), which is not electroactive. During orientation and as the polymer chains straighten, a polymorphic transition of the α-phase to the electroactive conformation -TT- (β-phase) occurs. Dipoles appear in the polymer volume, orienting and causing the appearance of the piezoelectric properties with the application of the electric field. In addition to the phase transition, orientation increases the polymer material’s electric strength [13], making it possible to raise the orienting electric field intensity and the material piezoresponse [14].

In the overwhelming majority of works devoted to the PVDF film orientation, the researchers focus their attention primarily on selecting the technological process parameters (degree and temperature) in stretching, which ensures the most complete transition of the *α*-phase [15,16,17,18] to the β-phase and studying the crystallinity degree and the relaxation processes. As a rule, a PVDF homopolymer is considered, and to obtain films with a high level of electroactive properties, they are uniaxially oriented at temperatures of 60–90 °C with a stretching ratio of 3–4 [14,19] and subsequent annealing at a temperature of 120 °C [20,21]. Despite the fact that a number of works show the influence of the polymer supramolecular structure phase composition on the material piezoelectric response [22,23,24], the literature practically misses any information on the orientation the technological process influence on the response magnitude. While it is important for further practical application of the films obtained. In addition, the literature poorly covers the aspect of the amorphous phase influence on the piezoelectric response magnitude, where the reversible transitions “paraelectric → ferroelectric” are quite possible during the mechanical stretching [25].

This paper considers the vinylidene fluoride copolymer with 6 mol% tetrafluoroethylene (VDF-TFE). Compared to other better-studied VDF copolymers, this material has a few advantages, making it possible to simplify the technological process in obtaining the polarized films. Unlike, for example, the PVDF homopolymer, it dissolves in a larger number of solvents and has lower melting points, facilitating the film formation process. Unlike the VDF/TrFE copolymer, it has a significantly lower cost. At the same time, there are significantly fewer works devoted to studying the mechanical treatment effect and thermal history of the VDF/TFE on its structure and electrophysical properties. Paper [26], for example, considers a VDF copolymer with 30 mol% TFE; however, only the stretching influence on the material’s crystallinity degree and dielectric properties is analyzed.

This paper applies the methods of scanning electron microscopy, piezoresponse force microscopy, differential scanning calorimetry, and IR spectroscopy to study the uniaxial orientation temperature influence on the structure and electroactive properties of the extruded VDF/TFE copolymer films. In addition, it discusses the molecular and supramolecular mechanisms of such orientation.

## 2. Results

This paper discusses films made from commercial VDF-TFE (HaloPolymer, Kirovo-Chepetsk, Russia). These films were produced by extrusion and then subjected to uniaxial orientation and uniaxial stretching at temperatures of 20, 40, 50, and 60 °C. After uniaxial stretching, the samples were polarized by the contact method at room temperature. The thickness of the samples under consideration was approximately 30 microns.

### 2.1. Electrical Strength Research

**Figure 1:**
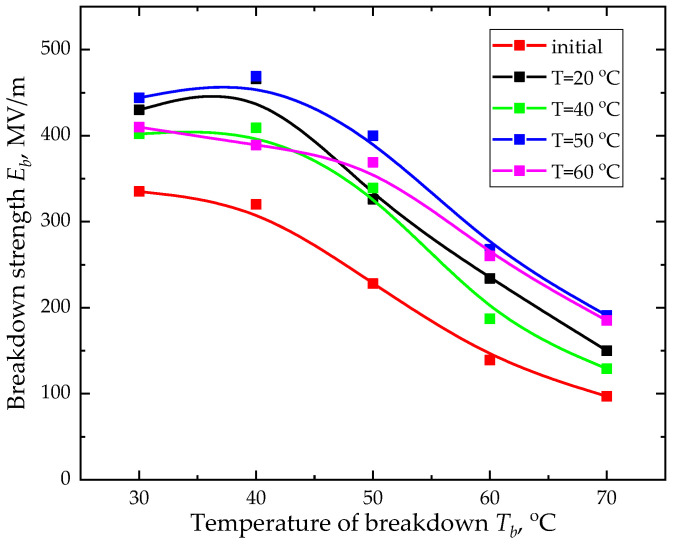
Electrical strength temperature dependence.

The orientation stretching temperature provides the strongest effect on the electrical strength dependence on the breakdown temperature. The electrical strength temperature dependence for the studied samples is not linear (Figure 1).

Most likely, this type of temperature dependence is associated with the structure alteration in stretching. It is found that orientation stretching increases the electrical strength value, thereby allowing polarization saturation to be achieved.

This paper notes that increasing the test temperature up to 40 °C leads to the growing electrical strength of the oriented samples. With the further increase in temperature, the electrical strength decreases. Such behavior of the electrical strength temperature dependence could be explained by the α_c_ relaxation process being characteristic for the vinylidene fluoride copolymers, which, according to the literature data [27,28], lies in the temperature range of 40–50 °C. The α_c_-process is associated with alteration in the molecular mobility of the polymer amorphous phase and the segmental motion of chains in the crystallites. At temperatures above the α_c_-relaxation, local overheating appears due to the energy dissipation, which reduces the electrical material strength.

### 2.2. Contact Polarization (Measuring the Macroscopic Piezoelectric Coefficients by the Berlincourt Method)

The piezoelectric coefficient dependence on the uniaxial stretching temperature is expressed to a lesser extent. The results obtained (Table 1) demonstrate that the *d*_33_ value dependence on the sample stretching temperature is non-monotonic.

Uniaxial orientation stretching provides for an increase in the piezoelectric coefficient from 5 pC/N to 20 pC/N, which allows us to use polarized film as an active element in sensors and actuators.

The piezoelectric coefficient maximum value is achieved at the stretching temperature of 20 °C. At the orientation temperature of 40 °C, the *d*_33_ value passes through its minimum: *d*_33_ is 13% less than the piezoelectric coefficient at the stretching temperature of 20 °C.

The piezoelectric coefficient values are also computed from the PFM data.

### 2.3. Piezoresponse Force Microscopy

**Figure 2:**
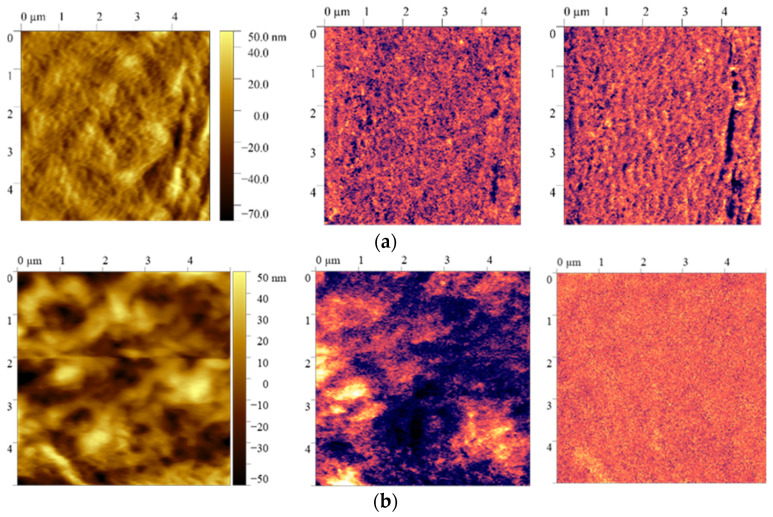
PFM data for a film stretched at 20 °C before (**a**) and after (**b**) polarization.

The piezoresponse force microscopy method is used to obtain distribution patterns of the vertical and lateral piezoresponse signals and compute the local piezoelectric coefficient values before and after film polarization. The data are presented in Table 2 and Figure 2.

It follows from the piezoresponse force microscopy data that the films after polarization have the most pronounced vertical piezoresponse signal, and their local piezoelectric coefficient value increases from 5 pm/V to 21 pm/V (film stretched at 20 °C).

**Figure 3:**
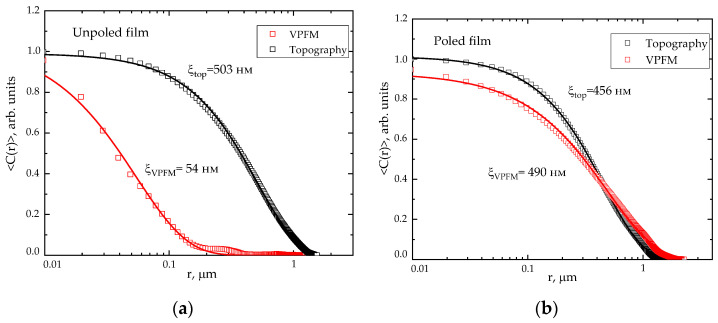
Autocorrelation function for topography and ferroelectric domains in the VDF/TFE film stretched at 20 °C showing differences in the correlation length for the unpolarized (**a**) and polarized (**b**) films.

As can be seen from the data in Figure 3, the vertical piezoelectric response contrast changes after polarization and consequently changes the ferroelectric domains’ size. The observed effect is characteristic of all the samples. To compute the ferroelectric domain sizes in the film before and after polarization, an autocorrelation function is constructed (Figure 3).

The characteristic domain size ξ is found by computing the correlation function of the vertical piezoresponse signal distribution depending on the *r* coordinate:

(3) Cr=A·exp−rξ2h,

where *A* is the constant; *r* is the distance from the central peak (nm) determined from the autocorrelation function image; ξ is the average value of the domain size (nm); and h (0 < h < 1) is the parameter [29,30].

Figure 3 presents an example of the autocorrelation function for a film oriented at 20 °C, showing the difference in correlation length of the topographic parameter *ξ_top_* and the size of the ferroelectric domain *ξ_VPFM_*. It is found that the domain size increases significantly after polarization from 54 nm to 490 nm. The measured domain size appears to be an order of magnitude higher than the characteristic crystallite size of the ferroelectric β-phase (*l*_001_ = 10 nm) [31], which indicates the contribution of the amorphous phase chains to the mechanism of ferroelectric domains formation in the VDF/TFE films.

An increase in the ferroelectric domain size of the oriented film after polarization could be explained by the contribution of the amorphous component chains. The macromolecule regions of the crystallite end surfaces form the intermediate ordering phase [26]. Therefore, when loaded along the drawing axis, these regions could lose mobility and become a part of the crystallite. If in the oriented state, the copolymer crystalline phase exists in both the ferroelectric and paraelectric forms, the external mechanical stress could initiate local reversible “paraelectric → ferroelectric” transitions.

### 2.4. Differential Scanning Calorimetry

It is natural to assume that the dependence of the VDF/TFE copolymer film’s electroactive properties on the stretching temperature is connected to an alteration in its structure during this process.

**Figure 4:**
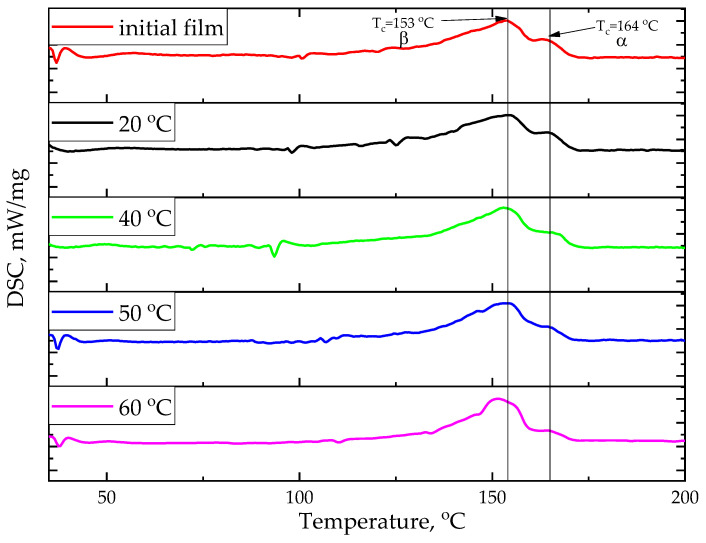
DSC curves of the first heating.

Using differential scanning calorimetry makes it possible to analyze the samples’ structure after stretching. Three endothermic peaks corresponding to the crystallite melting are found on the curves of the first heating (Table 3). This indicates the presence of several crystalline phases in the sample. For the samples stretched at 20 °C and 50 °C, the peak corresponding to 145 °C is weakly expressed (Figure 4).

This paper notes that uniaxial stretching insignificantly influences the crystallinity degree of the studied samples. All films are characterized by the presence of several crystalline phases. Films stretched at 20 °C and 50 °C demonstrate maximum crystallinity.

It is also worth mentioning the existence of a long, low-temperature “tail” in the crystallization processes, which could indicate the crystallite defectiveness formed during extrusion and the subsequent stretching.

### 2.5. IR Fourier Spectroscopy

**Figure 5:**
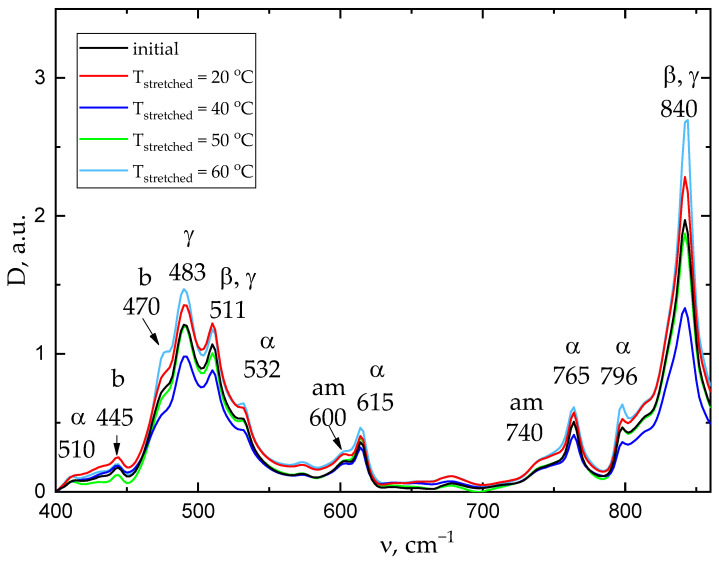
IR Fourier spectroscopy transmission spectra.

The measured IR spectra are shown in Figure 5. The electroactive and *α*-phases content changes slightly (up to 3.5%) and non-monotonically depending on the stretching temperature (Table 4). In general, phase composition also changes slightly as a result of mechanical stretching. The sample stretched at a temperature of 40 °C has the lowest proportion of the ferroelectric phase. It is also characterized by the minimum piezoelectric coefficient among the stretched samples.

### 2.6. Density Measurement by the Hydrostatic Weighing Method

The sample density after stretching is measured by the hydrostatic weighing method (Table 5).

The paper shows that uniaxial orientation stretching significantly increases the film density. In this case, the density value depends nonlinearly on the stretching temperature. The maximum density value (1.94 g/cm^3^) is typical for a film oriented at 40 °C. It was shown previously that the piezoelectric coefficient at this stretching temperature was minimal (16 pC/N).

### 2.7. Scanning Electron Microscopy

The presented data demonstrates that the crystallinity degree and density of the uniaxially oriented film (with a generally non-monotonic nature of dependences with alteration in the stretching temperature) are changing antibatically and reach the extreme point at the stretching temperature of 40 °C. This confirms the previously advanced assumption that the structure of the film oriented at a temperature of 40 °C differs from its structure formed at the other stretching temperatures.

**Figure 6:**
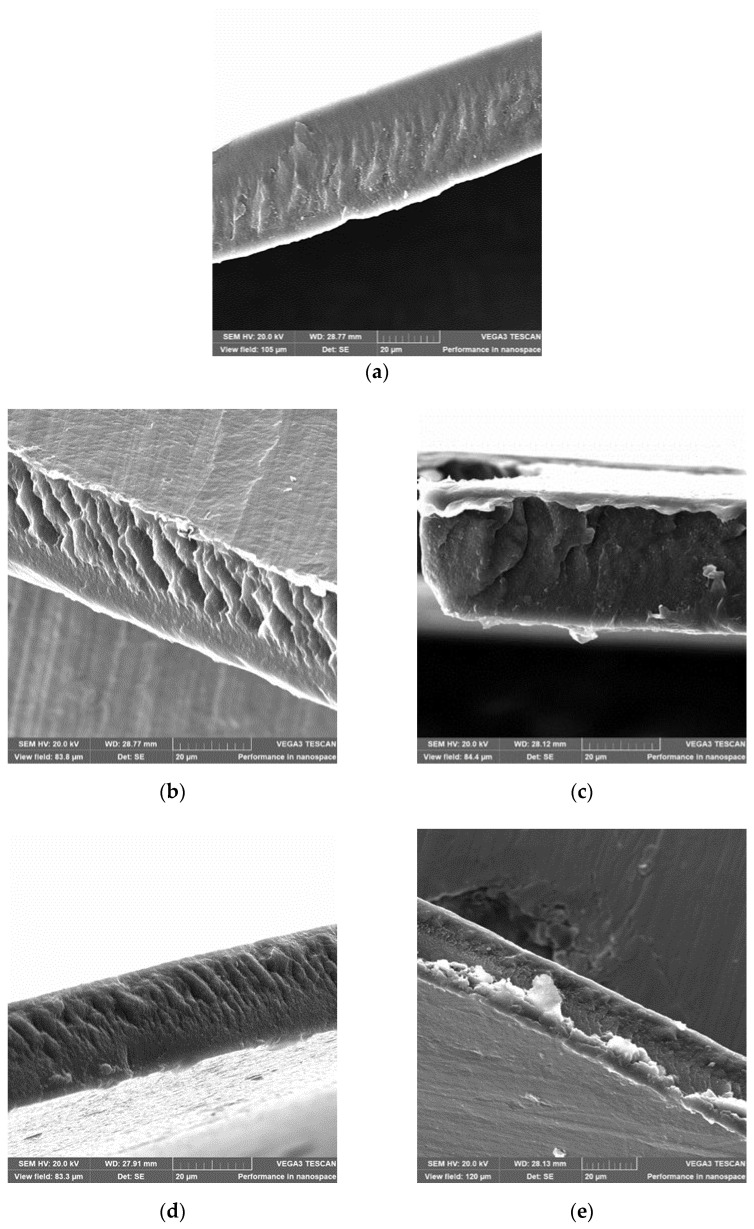
Micrographs of cold cleavages of the films stretched at different temperatures: (**a**) initial film, (**b**) temperature of stretching 20 °C, (**c**) temperature of stretching 40 °C, (**d**) temperature of stretching 50 °C, and (**e**) temperature of stretching 60 °C.

The SEM micrographs of “cold” cleavage of the films oriented at different temperatures could be considered as an indirect confirmation of this conclusion (Figure 6).

The presented results show that surfaces of all the studied samples contain layers inclined at an angle to the surface, which are characteristic of the uniaxially oriented polymers [32]. Probably, the shear bands are filled with the highly dispersed oriented fibrilized material. The fibrillary structure characteristic picture is mostly indicative of the samples stretched at temperatures of 20 °C and 50 °C, which have the highest piezoelectric coefficients.

## 3. Discussion

The mechanical uniaxial stretching results in compacting the material structure, as evidenced by an increase in density by 11–21%. The increase in density probably means that the fibrils formed during the uniaxial orientation are compacted under the compressive forces acting in the transverse direction. Under such conditions, the deformation mode changes; i.e., the lamellas spreading turns into the interlamellar shear. The amorphous component is compressed between the lamellae, which leads to a decrease in the material’s free volume and, consequently, to an increase in its density. Apparently, the orientation of the amorphous phase chains occurs during the uniaxial stretching. This in turn causes a decrease in the amorphous component mobility, which contributes to the formation of the larger domains due to the transition of the metastable paraelectric phase regions to the ferroelectric state under the mechanical stress action. As a result, the domain size increases from 40 to 350 nm in the stretched samples under the electric field action. 

In addition, an increase in the material electrical strength by ~20% after the mechanical stretching is identified. This could be explained by the fact that the polymer free volume decreases in the acting compressive stress field, and the mobility of the injected charges consequently decreases [30]. Since counter motion of the oppositely charged traps determines the electrical strength magnitude, a decrease in mobility leads to an increase in the field strength at which an electrical breakdown occurs. As a result, the material polarization efficiency increases, and the stretched films acquire piezoelectric coefficients *d*_33_ at the level of 16–20 pC/N, which is comparable with the β-PVDF piezoelectric coefficient values. The obtained piezoelectric response values make it possible to manufacture devices based on the VDF-TFE copolymer film.

In this case, the *d*_33_ value depends on the crystallinity degree and the ferroelectric phase proportion. The sample stretched at a temperature of 40 °C has the lowest piezoelectric coefficient (16 pC/N). It is also characterized by the minimum ferroelectric phase proportion and crystallinity degree among the stretched samples. The samples stretched at the temperatures of 20 °C and 50 °C have the highest piezoelectric coefficients (20 and 19 pC/N, respectively). They are also characterized by the highest crystallinity degree.

## 4. Materials and Methods

This paper studied VDF/TFE copolymer films obtained by melt extrusion. Fluoroplastic powder F2m grade B (HaloPolymer, Kirovo-Chepetsk, Russia) was used to receive the film; extrusion was performed by the IIPT SPE “Plastic”. The initial film thickness was 30 μm.

### 4.1. Uniaxial Stretching Technique

Uniaxial stretching was carried out at four different temperatures of 20, 40, 50, and 60 °C, and the stretching ratio was 3. It was noted that the stretched film thickness differed from that of the original film by few microns, indicating the non-trivial material texturing process.

A laboratory machine for polymer film orientation (DACA Instrument, Santa Barbara, CA, USA) was used to stretch the extruded film.

The required deformation value was determined by adjusting speeds of the receiving and feeding rollers. The film was heated in orientation on a flat heater, along which the film was displaced during rewinding.

### 4.2. Contact Polarization

After uniaxial stretching, the samples were polarized by the contact method at room temperature for 30 s. The polarizing field voltage was 300 MV/m.

Contact polarization was carried out using a laboratory bench, which schematic representation is provided in Figure 1. In addition, the bench was introduced to measure electrical strength of the samples.

The samples were placed in the Vaseline oil bath, and the 2.5 mm diameter aluminum electrode was positioned on the sample top. The polarizing voltage was supplied to the sample from the high-voltage electrode.

### 4.3. Research Methods

#### 4.3.1. Macroscopic Piezoelectric Coefficient *d*_33_ Measurement

Macroscopic piezoelectric coefficients *d*_33_ values were measured by the quasi-static Berlincourt method using the YE2730A *d*_33_-meter (Sinocera Piezotronics, Yangzhou, China).

#### 4.3.2. Electrical Strength Measurement

**Figure 7:**
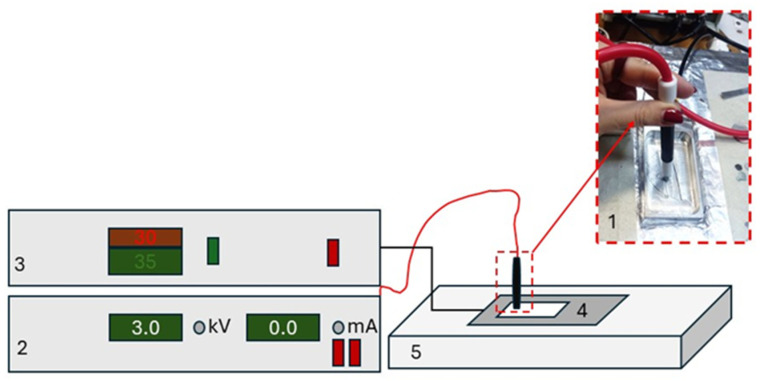
Laboratory bench for the contact polarization: 1—high-voltage electrode; 2—voltage unit; 3—temperature control unit; 4—Vaseline oil bath; and 5—heating table.

Electrical strength temperature dependences were computed for all the samples under study. The measurements were taken in the temperature range from 20 °C to 110 °C and the breakdown field characteristic values at the polarization temperature (30 °C). Electrical strength was measured using the laboratory bench (Figure 7) in the Vaseline oil bath to avoid breakdown in the air.

The breakdown field values at each temperature were obtained using the two-parameter Weibull model [1,33,34,35,36,37], according to which a statistical set of a large number of field values E_b_ could be described by the following function:

(4) $$F\left(x\right)=1-\exp[-{\left(\frac{x}{\alpha }\right)}^{\beta_{b}}],$$

where x is the breakdown field current value; α is a certain characteristic field, at least 63.2% of the test samples are broken down; and the $\beta_{b}$ parameter characterizes the breakdown field dispersion relative to the average value.

#### 4.3.3. Piezoresponse Force Microscopy

Ferroelectric properties and morphology alteration were studied by the scanning probe microscopy (SPM) methods using the NTEGRA Prima atomic force microscope (NT-MDT SI, Zelenograd, Russia) with cantilevers with the FMG01/Pt platinum conductive coating (Tipsnano, Tallinn, Estonia). Experimental data were processed using the Gwiddion 2.67 software (Czech Metrology Institute, Brno, Czech Republic).

SPM was used to obtain the film surface topographies and compute the root-mean-square (RMS) roughness values for the samples stretched at different temperatures. In the piezoresponse force microscopy mode, vertical and lateral piezoresponse signal distribution maps were obtained, and the local piezoelectric coefficients *d*_33_ were computed before and after contact polarization. HA_HR/Pt conducting cantilevers (NT-MDT SI, Zelenograd, Russia) with the strength constant of 17 N/m were used to measure the local piezoelectric coefficients. The local piezoelectric coefficients were determined by direct measurement of deformation when applying the alternating voltage from 0 to 10 V at the fixed frequency of 890 kHz. Measurements were taken at two points: near the breakdown point and at a certain distance. The piezoelectric coefficients *d*_33_ values were computed from the Mag1n signal:

(5) $$d_{33}^{*}=\frac{\mathrm{Mag}\;1n}{U\cdot Q},$$

where U is the voltage supplied in scanning (B); Q is the quality factor shows the quality of the oscillatory system; and the Mag 1n signal corresponds to the sample deformation (pm).

The single-crystal lithium niobate with the known value of *d*_33_ = 18 pm/V was chosen as the calibration sample.

#### 4.3.4. Scanning Electron Microscopy

The film surface morphology was studied using scanning electron microscopy (SEM) with the TESCAN VEGA3 microscope (TESCAN Brno s.r.o., Brno, Czech Republic). Images of the cold cleavage were obtained using a secondary electron detector (SE). Before scanning, a thin layer (about 20 nm) of the conductive coating (gold) was deposited on the sample’s surface to prevent charge spreading over the dielectric surface. The metal coating was deposited using the SPI-MODULE Sputter Coater magnetron sputtering device (SPI supplies, West Chester, PA, USA). Micrographs of the cold cleavage were also obtained for the films stretched in different modes. The films were cooled in liquid nitrogen for 5 min. Before scanning, the samples were mounted onto special vertical holders using carbon tape, allowing us to remove the ends of the film.

#### 4.3.5. Differential Scanning Calorimetry

The samples’ thermal properties and crystallinity degree were determined by differential scanning calorimetry (DSC) on the NETZSCH DSC 204F1 Phoenix device (NETZSCH-Gerätebau GmbH, Selb, Germany). The samples were placed for measurement in the aluminum crucibles, and they were heated in the temperature range of 25–200 °C at a heating rate of 2 K/min in the argon medium.

The DSC curves are applied to compute the film crystallinity degree, considering the melting enthalpy of all crystallizing comonomers and their ratios [38]:

(6) $$\chi_{c}=\frac{∆H_{m}}{\omega_{VDF}\cdot ∆H_{PVDF}^{\emptyset }+\omega_{TFE}\cdot ∆H_{PTFE}^{\emptyset }},$$

where ∆H_m_ is the film melting enthalpy; $\omega_{VDF}\;$ is mol% of VDF monomer;$∆H_{PVDF}^{\emptyset }$ is the melting enthalpy of the fully crystalline PVDF, which is 104.5 J/g [39,40]; $\omega_{TFE}$-is mol% of TFE monomer; and $∆H_{PTFE}^{\emptyset }$-is the melting enthalpy of the fully crystalline PTFE (82 J/g).

#### 4.3.6. Fourier Transform Infrared Spectroscopy and Computation Based on It

Phase composition of the experimental samples was determined by Fourier transform IR spectroscopy. The transmission coefficients were measured using the PerkinElmer 1760X FTIR IR Fourier spectrometer (PerkinElmer Inc., Waltham, MA, USA) in the wavelength range of 400–4000 cm^−1^.

The spectra were processed and decomposed in the Systat PeakFit 4.12.00 (Systat Software Inc., San Jose, CA, USA) software program. The film phase composition (electroactive phase and α-phase proportions) was computed according to the method provided in [41].

The electroactive phase proportion was determined by the following formula:

(7) $$F_{\mathrm{EA}}=\frac{I_{\mathrm{EA}}}{\left(\frac{K_{840}}{K_{763}}\right)I_{763}+I_{\mathrm{EA}}}\cdot 100\%,$$

where $I_{763}$ is the amplitude at the 763 cm^−1^ peak (alpha-phase peak); $I_{EA}$ is the amplitude at the 840 cm^−1^ peak (electroactive phase peak (β- and γ-phases)); and $K_{840\;}=7.7\;\times \;10^{4}\;\frac{{\mathrm{cm}}^{2}}{\mathrm{vol}},K_{763}=6.1\;\times \;10^{4}\;\frac{{\mathrm{cm}}^{2}}{\mathrm{mol}}$ are the absorption coefficients at the corresponding wave values.

The $F_{\alpha }\;$ alpha phase proportion was computed assuming that the material contained 3 phases (α, β, and γ):

(8) $$F_{\alpha }=100\;{-F}_{\mathrm{EA}}$$

#### 4.3.7. Density Measurement by the Hydrostatic Weighing

Density was measured by the hydrostatic weighing method according to ISO 1183-1:2025 [42] “Plastics—Methods for determining the density of non-cellular plastics—Part 1: Immersion method, liquid pycnometer method and titration method”. Weighing was carried out in liquid with the known density, i.e., in distilled water. The M-ER 123 ACFJR “SENSOMATIC” TFT analytical scales (Mertech, Seoul, Republic of Korea) with the weighing accuracy of up to 0.0001 g were used in weighing.

## 5. Conclusions

This paper examines the effect of uniaxial orientation stretching on the piezoelectric properties and electrical strength of VDF/TFE copolymer film. It found that orientation stretching increases the electrical strength, thereby allowing the achievement of polarization saturation. At the same time, electrical strength decreases at temperatures above 40 °C, where the relaxation *α_c_*-process appears, leading to an increase in the cooperative mobility and local overheating. This determines technological modes of film polarization and limits operating conditions of the functional devices based on it.

Uniaxial orientation stretching contributes to an increase in the piezoelectric coefficient *d*_33_ from 5 pC/N to 16–20 pC/N. Apparently, this is associated with the orientation of the amorphous phase chains, which leads to a decrease in their mobility. In addition, it promotes the formation of the larger domains due to the transition of the metastable paraelectric phase regions to the ferroelectric phase under the mechanical stress action. This paper shows that the domain size increases significantly from 40 to 350 nm after polarization.

The obtained results indicate the importance of the amorphous phase contribution to the formation of the polymer material’s piezoelectric properties.

In addition, this paper shows that in the case of the film contact polarization at 30 °C, the piezoelectric coefficient *d*_33_ value measured by the quasi-static Berlincourt method is almost equal to the *d*_33_ value of the commercial PVDF homopolymer (20 pC/N) polarized at 80 °C. This makes it possible to use polarized VDF-TFE film as an active element in sensors and actuators.

## Figures and Tables

**Table 1:** Material piezoelectric properties.

Stretching Temperature, °C	*E_b_*_(30°C),_ MV/m	*d*_33_, pC/N
Initial film	335	5
20	430	20
40	402	16
50	440	19
60	410	18

**Table 2:** Piezoelectric response force microscopy data.

Stretching Temperature, °C	RMS, nm		*d*_33_*^loc^* pm/V		*ξ_VPFM_*, nm	
	Unpoled	Poled	Unpoled	Poled	Unpoled	Poled
Initial film	68	30	4	11	42	183
20	36	40	4,5	21	54	490
40	28	55	5	19	21	350
50	45	59	4,5	23	37	270
60	42	33	4	17	28	380

**Table 3:** Crystallinity degree computed from the DSC data.

Stretching Temperature, °C	Melting Enthalpy ΔHm, J/g	Crystallinity Degree χ_c_, %	Melting Temperature	
			β	α
Initial	44.0	42.7	153.5	162.8
20	47.7	46.2	154.1	164.0
40	43.5	42.2	153.1	165.1
50	48.7	47.2	153.1	164.0
60	46.7	45.3	151.4	164.2

**Table 4:** Results of the IR Fourier spectroscopy study.

T_stretching_, °C	Initial	20	40	50	60
Electroactive phase proportion, %	80.6	78.1	77.0	80.0	80.5
*α*-phase proportion, %	19.4	21.9	23.0	20.0	19.5

**Table 5:** Material density measurements.

T_stretching_, °C	Initial	20	40	50	60
ρ, g/cm^3^	1.60	1.78	1.94	1.80	1.82

## Data Availability

The original contributions presented in this study are included in the article. Further inquiries can be directed to the corresponding author(s).
